# Simultaneous quantitation of multiple myeloma related dietary metabolites in serum using HILIC-LC-MS/MS

**DOI:** 10.29219/fnr.v67.9135

**Published:** 2023-07-28

**Authors:** Mo Wang, Rui Zhang, Shunli Zhang, Xiaojie Zhou, Yichuan Song, Qingtao Wang

**Affiliations:** 1 Department of Clinical Laboratory, Beijing Chaoyang Hospital, Beijing Center for Clinical Laboratories, The Third Clinical Medical College of Capital Medical University, Beijing, P.R. China; 2 Department of Clinical Laboratory, Beijing Chaoyang Hospital, The Third Clinical Medical College of Capital Medical University, Beijing, P.R. China; 3 Department of Clinical Laboratory, Beijing Chaoyang Hospital, Capital Medical University, Beijing, P.R. China

**Keywords:** metabolites, liquid chromatography tandem mass spectrometry, multiple myeloma, diet

## Abstract

**Background:**

Recent studies from targeted and untargeted metabolomics have consistently revealed that diet-related metabolites, including carnitine (C0), several species of acylcarnitines (AcyCNs), amino acids, ceramides, and lysophosphatidylcholines (LPCs) may serve as potential multiple myeloma (MM) biomarkers. However, most of these approaches had some intrinsic limitations, namely low reproducibility and compromising the accuracy of the results.

**Objective:**

This study developed and validated a precise, efficient, and reliable liquid chromatography tandem mass spectrometric (LC-MS/MS) method for measuring these 28 metabolic risk factors in human serum.

**Design:**

This method employed isopropanol to extract the metabolites from serum, gradient elution on a hydrophilic interaction liquid chromatographic column (HILIC) for chromatographic separation, and multiple reaction monitor (MRM) mode with positive electrospray ionization (ESI) for mass spectrometric detection.

**Results:**

The correlation coefficients of linear response for this method were more than 0.9984. Analytical recoveries ranged from 91.3 to 106.3%, averaging 99.5%. The intra-run and total coefficients of variation were 1.1–5.9% and 2.0–9.6%, respectively. We have simultaneously determined the serological levels of C0, several subclasses of AcyCNs, amino acids, ceramides, and LPCs within 15 min for the first time.

**Conclusion:**

The established LC-MS/MS method was accurate, sensitive, efficient, and could be valuable in providing insights into the association between diet patterns and MM disease and added value in further clinical research.

## Popular scientific summary

- Diet related metabolites of serum are potential biomarkers for multiple myeloma (MM).
- We present an efficient and precise LC-MS/MS method for simultaneous quantification of these 28 metabolic risk factors in human serum within 15 min.

Multiple myeloma (MM) is a kind of malignant neoplasm characterized by abnormal proliferation of clonal plasma B-cells in the bone marrow (1), and it ranks as the second most common hematologic malignancy in many countries (2, 3). Although MM survival has improved due to the development of novel diagnostic techniques and treatment strategies, it is still considered primarily incurable because of the recurrent relapsing disease course (4). Moreover, since knowledge of MM etiology is insufficient and most definite risk factors of MM are unmodifiable, few prevention strategies can be implemented for this refractory disease.

In recent years, much evidence raised the possibility that MM pathogenesis was influenced by dietary patterns (5–7). Since the food can supply more than 27,000 chemicals and extra thousands of gut microbiota-derived and host-derived metabolites, the composition, concentration, and functions of the serum circulating metabolites are dynamic and affected by diet properties (8, 9). For example, some species of serum amino acids, lipids such as ceramides and lysophosphatidylcholines (LPCs), secondary metabolites such as acylcarnitines (AcyCNs) may be induced by increasing the high-protein or overall lipid load from the diet (10–12). At the same time, metabolomics analysis of MM has confirmed that the content of these diet-derived endogenous metabolites was significantly altered: higher levels of branched-chain amino acids (BCAAs), aromatic amino acids (AAAs), Glutamic acid (Glu) and decreased levels of Glutamine (Gln), several species of AcyCNs have been exhibited in MM patients at diagnosis compared with the stage of achieving complete remission (13–16); higher levels of LPCs [16:0 (palmitoyl) and LPC 18:0 (stearoyl)], as well as lower values of ceramides, suggested the potential of MM event compared with the control set (17–19). These consistent results documented that alterations of serum metabolite may presage the onset of MM. Unfortunately, metabolomics studies focused on biomarker discovery, and both precision and accuracy were unsatisfactory. Additional precise and reliable methods and validation procedures on independent external populations are required to assess whether diet-related compounds are useful as clinical biomarkers. Numerous liquid chromatography tandem mass spectrometry (LC-MS/MS) based measurements for the analytes have been described in the literature (20–23). However, most of these methods quantitated only one or a couple of the metabolites of interest. Establishing an accurate, simple, and reliable approach for simultaneously measuring all compounds is essential.

The objective of this research was to establish an accurate, simple, sensitive, and efficient LC-MS/MS method for simultaneous determination of diet-related MM metabolic factors, including C0, 13 AcyCNs, eight amino acids, three ceramides, and three LPCs in human serum. This assay requires a minimal volume of serum samples, using the protein precipitation method for quick sample preparation, and separates all the chemicals in 15 min using a hydrophilic interaction liquid chromatography (HILIC) column. The established and validated method should be useful for evaluating the association among the dietary metabolites, diet patterns, and etiology of MM. Furthermore, this assay may assist in the identification of at-risk individuals and monitoring response to chemoradiation therapies of MM by providing more information over traditional clinical markers.

## Experimental method

### Materials and reagents

Analytical grade methanol and isopropanol were obtained from Thermo Fisher Scientific (Newark, DE, USA). Sigma-Aldrich (St. Louis, MO, USA) supplied standards of amino acids [tyrosine (Tyr), phenylalanine (Phe), tryptophan (Trp), valine (Val), isoleucine (Ile), leucine (Leu), Glu, Gln], free C0, most of AcyCNs [C2 (acetyl), C3 (propionyl), C4 (butyryl), C5 (valeryl), C6 (hexenoyl), C8 (octanoyl), C10 (decanoyl), C10:1 (decenoyl), C14 (tetradecanoyl), C16 (hexadecanoyl), C18 (octadecanoyl)], ammonium acetate and acetic acid. Standards of dodecanoycarnitine (C12), oleoylcarnitine (C18:1), three ceramides [C16 (d18:1/16:0), C18 (d18:1/18:0), C24 (d18:1/24:0)], standards of LPC 16:0 (palmitoyl), LPC 18:0 (octadecanoyl), LPC 18:1 (oleoyl), internal standards of ceramide (C16-D31, C18-D7, C24-D7) and LPC 19:0 (nonadecanoyl) were attained from Avanti Polar Lipids (Alabaster, AL). We obtained isotopically labeled internal standards for C0, 13 AcyCNs, and eight amino acids from C/D/N Isotopes Inc. (Quebec, Canada).

### Calibrators and internal standards preparation

In accordance with the concentration ranges of the metabolites in serum, a series of five-level calibrator working solutions were prepared (20, 24, 25). Accurately weighed standards of these compounds were transferred to a glass vial and dissolved in isopropanol with 0.1% acetic acid to make the stock standard. As shown in Table 1, four calibration standards with varying concentrations (S2–S5) were formed by diluting the mix stock standard (S1). A working solution of mixed internal standards for the analytes was prepared by dissolving the powders to achieve a universal concentration at S3. All prepared solutions were aliquoted and kept at −80°C in hermetically sealed glass vials prior to analysis.

**Table 1:** Standard concentrations (μmol/L) and internal standards of amino acids, ceramides, LPCs, C0 and AcyCNs

Metabolites	Species	S1	S2	S3	S4	S5	Internal standard
Amino acids	Val	21.51	107.55	215.11	322.66	430.22	Val-D8
	Ile	18.95	94.74	189.47	284.21	378.95	Ile-D10
	Leu	18.82	94.10	188.20	282.31	376.41	Leu-D2
	Tyr	13.97	69.85	139.71	209.56	279.41	Tyr-D2
	Phe	14.99	74.94	149.89	224.83	299.78	Phe-D2
	Trp	12.27	61.37	122.74	184.11	245.47	Trp-D3
	Glu	17.11	85.55	171.10	256.64	342.19	Glu-D5
	Gln	68.64	343.19	686.37	1029.56	1372.75	Gln-D5
Ceramides	C16	0.10	0.49	0.98	1.47	1.96	C16-D31
	C18	0.04	0.18	0.37	0.55	0.74	C18-D7
	C24	0.67	3.33	6.66	10.00	13.35	C24-D7
LPCs	16:0	10.46	52.29	104.58	156.87	209.16	LPC 19:0
	18:0	4.27	21.35	42.7	64.05	85.4	LPC 19:0
	18:1	2.27	11.37	22.75	34.12	45.50	LPC 19:0
C0 and AcyCNs	C0	9.31	46.53	93.05	139.58	186.10	C0-D3
	C2	1.02	5.10	10.21	15.31	20.41	C2-D3
	C3	1.05 × 10^-1^	5.25 × 10^-1^	1.05	1.57	2.10	C3-D3
	C4	4.32 × 10^-2^	2.16 × 10^-1^	4.32 × 10^-1^	6.49 × 10^-1^	8.65 × 10^-1^	C4-D3
	C5	9.10 × 10^-3^	4.55 × 10^-2^	9.10 × 10^-2^	1.37 × 10^-1^	1.82 × 10^-1^	C5-D3
	C6	9.69 × 10^-3^	4.85 × 10^-2^	9.69 × 10^-2^	1.45 × 10^-1^	1.94 × 10^-1^	C6-D3
	C8	4.04 × 10^-2^	2.02 × 10^-1^	4.04 × 10^-1^	6.06 × 10^-1^	8.08 × 10^-1^	C8-D3
	C10	7.42 × 10^-2^	3.71 × 10^-1^	7.42 × 10^-1^	1.11	1.48	C10-D3
	C10:1	1.37 × 10^-1^	6.85 × 10^-1^	1.37	2.06	2.74	C10-D3
	C12	3.20 × 10^-2^	1.60 × 10^-1^	3.20 × 10^-1^	4.80 × 10^-1^	6.40 × 10^-1^	C12-D3
	C14	8.43 × 10^-3^	4.22 × 10^-2^	8.43 × 10^-2^	1.26 × 10^-1^	1.69 × 10^-1^	C14-D3
	C16	2.72 × 10^-2^	1.36 × 10^-1^	2.72 × 10^-1^	4.08 × 10^-1^	5.44 × 10^-1^	C16-D3
	C18	7.30 × 10^-3^	3.65 × 10^-2^	7.30 × 10^-2^	1.09 × 10^-1^	1.46 × 10^-1^	C18-D3
	C18:1	9.05 × 10^-2^	4.53 × 10^-1^	9.05 × 10^-1^	1.36	1.81	C18-D3

### Samples preparation

Serum pools were gathered from the residual patient samples at the Department of Clinical Laboratory of Beijing Chaoyang Hospital for method development and validation. The calibrators, internal standard, and serums were defrosted, and then brought to room temperature. Aliquots of 10 μL calibrators or serums were precisely transferred into 2 mL tubes by 500 μL of isopropanol containing 0.1% acetic acid, and then added 10 μL of the internal standard solution through washing with 500 μL of isopropanol containing 0.1% acetic acid to each vial with an automatic diluter. All vials were shaken for 15 min and then centrifugation to protein precipitation. After transferring an aliquot of 300 μL supernatant to a different vial, it was evaporated in front of nitrogen. Then, the residue was dissolved in a total volume of 300 μL of the mobile phase and finally analyzed using LC-MS/MS.

### Instrumental analysis

Shimadzu Nexera-XR Series HPLC (Shimadzu, Kyoto, Japan) was used for chromatographic separation. Following sample preparation, with a Phenomenex Kinetex hydrophilic interaction liquid chromatography column (HILIC, 150 mm × 2.1 mm, 2.6 μm), 3 μL reconstituted samples were injected into the analysis system. The column was operated under gradient conditions with mobile phase A (water, containing 0.5% acetic acid and 10 mM ammonium acetate) and mobile phase B (methanol, containing 0.5% acetic acid). The gradient program consisted of: 0–0.5 min: from 10% A to 35% A, 0.5–5 min: 35% A, 5–5.5 min: from 35% A to 15% A, 5.5–10 min: 15% A, 10–10.5 min: from 15% A to 10% A, 10.5–15 min: 10% A. Throughout the analysis, the flow rate was maintained at 200 μL/min, and the temperatures of the auto-sampler and column oven were kept at 4 and 25°C, respectively.

MS/MS analysis was carried out on triple quadrupole linear ion trap 5500 tandem mass spectrometry (Sciex Applied Biosystems). The multiple reaction monitor (MRM) mode was utilized for the MS detection process, which was carried out using positive electrospray ionization (ESI). The scheduled detection process has been divided into two periods: the first ended at 4 min, and the second started then and continued to 10 min of the run. For the first period, the probe temperature and ion spray voltage, curtain gas, nebulizer gas, and collision gas were set at 400°C, 2,500 V, 25 psi, 60 psi, and low, respectively. For the second one, the corresponding source parameters were set at 550°C, 4,000 V, 30 psi, 70 psi, and medium. The dwell time was set at 50 ms. The liquid chromatography eluent was sent directly to the electrospray interface during the first 10 min, allowing the targeted analytes to be sequentially eluted, ionized, and detected by the system, then switched to the waste from 10 to 15 min to re-equilibrate the column.

### Method validations

The linearity of the calibration curve of each metabolite was evaluated based on the regression between peak area ratios (y) of the analyses to their internal standards and corresponding concentrations of them (x). To assess the recoveries of these metabolites, a serum was first spiked with two levels of mixed standard solutions, and then both spiked and unspiked serum samples were subjected to metabolites concentration analysis. Analytical recoveries were calculated as ratios of actual increasing concentration measurements to the expected values. Imprecisions were assessed by measurement of three replicates of three serum pools on 3 consecutive days. By diluting the lowest standard solution, the limits of quantification (LOQ) and detection (LOD) were also assessed. LOQ was defined as the concentrations that produced a signal-to-noise (S/N) ratio of 10, while the LOD was defined as the concentrations that generated a S/N ratio of 3.

## Results

### Method development and optimization

#### Optimization of MS/MS detection parameters

Under a variety of different conditions, the precursor and product ions of all the compounds were determined by injecting every standard into the triple quadrupole mass spectrometer using a syringe pump. The MS/MS scan was performed on all metabolites using both the positive and the negative ion modes, and the results showed that the positive ion mode produced higher signal strength. After then, the other settings of the MS/MS detection parameters were tuned to get the best separation and the highest detection specificity. To quantitate Leu and Ile, which are isobaric molecules, product ions of m/z 43 and m/z 69 were used respectively. Before calculating peak area ratios of amino acids to internal standards, Leu and Ile peak areas were corrected according to our previous study (26) as follows: A_Leu_, _corrected_ = A_m/z 132→m/z 43_ – 0.02 × A_m/z 132→m/z 69_, and A_Ile_, _corrected_ = A_m/z 132→m/z 69_ – 0.06 × A_m/z 132→m/z 43_. Their corresponding internal standards did not require such adjustments. Table 2 shows the parent ions, product ions, and the optimized parameters, including declustering potential (DP), entrance potential (EP), collision energy (CE), collision exit potential (CXP), and dwell times used for monitoring the metabolites. Two periods were performed to detect 28 metabolites: eight amino acids and three ceramides were monitored during the first section, and the remaining analytes, including LPCs, C0, and AcyCNs, were tested in the second period.

**Table 2:** Optimized LC-MS/MS conditions for MRM chromatographic acquisition of amino acids, ceramides, LPCs, C0 and AcyCNs

Metabolites	Species	Period	Retention time (min)	Ion transition (m/z)	DP (psi)	EP (psi)	CE (psi)	CXP (psi)
Amino acids	Val	I	2.62	118.0→72.0	40	8	15	8
	Ile	I	2.56	132.0→69.0	40	6	23	10
	Leu	I	2.56	132.0→43.0	50	8	14	10
	Tyr	I	2.42	182.0→136.0	55	8	18	15
	Phe	I	2.52	166.1→120.0	40	8	22	25
	Trp	I	2.52	205.0→188.0	50	8	14	20
	Glu	I	2.73	148.1→84.0	30	8	24	25
	Gln	I	3.08	147.1→84.0	40	8	13	29
Ceramides	C16	I	1.84	538.4→264.4	85	8	34	9
	C18	I	1.83	566.5→548.4	77	7	18	21
	C24	I	1.81	651.0→632.6	110	7	16	25
LPCs	16:0	II	6.52	496.2→184.0	120	8	35	17
	18:0	II	6.39	525.1→184.0	100	9	36	14
	18:1	II	6.45	522.3→184.0	120	10	47	13
C0 and AcyCNs	C0	II	6.97	162.1→85.0	80	8	27	9
	C2	II	7.32	204.2→85.0	120	10	28	25
	C3	II	7.14	218.1→85.0	110	8	25	10
	C4	II	6.88	232.1→85.0	120	8	26	13
	C5	II	6.93	246.0→85.0	110	8	27	9
	C6	II	6.37	260.1→85.0	130	8	27	9
	C8	II	5.97	288.1→85.0	110	6	27	9
	C10	II	5.7	316.1→85.0	110	8	29	10
	C10:1	II	5.71	314.2→85.0	140	6	30	13
	C12	II	5.55	344.5→85.0	100	7	31	10
	C14	II	5.44	372.2→85.0	150	7	31	10
	C16	II	5.34	400.2→85.0	100	8	32	10
	C18	II	5.28	428.2→85.0	200	8	33	10
	C18:1	II	5.28	426.3→85.0	140	8	33	13

#### HPLC conditions

**Fig. 1:**
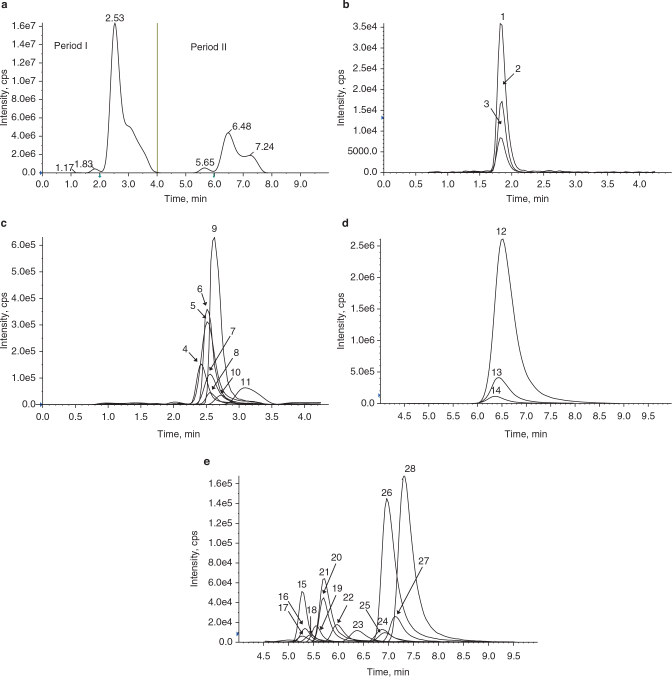
Representative LC-MS/MS chromatogram of 28 metabolites extracted from a human serum pool. (a) Total ion chromatogram of 28 metabolites in two periods under the gradient elusion. (b) Chromatogram of three ceramids in period I. 1. C16 ceramide; 2. C18 ceramide; 3. C24 ceramide. (c) Chromatogram of eight amino acids in period I. 4. Tyr; 5. Phe; 6. Trp; 7. Leu; 8. Ile; 9. Val; 10. Glu; 11. Gln. (d) Chromatogram of three LPCs in period II. 12. LPC 16:0; 13. LPC 18:0; 14. LPC 18:1; (e) Chromatogram of C0 and 13 AcyCNs in period II. 15. C18:1; 16. C18; 17. C16; 18. C14; 19. C12; 20. C10; 21. C10:1; 22. C8; 23. C6; 24. C4; 25. C5; 26. C0; 27. C3; 28.C2.

In order to attain good separation and high-resolution peaks, mobile phase constitution with different gradients, flow rates, types of separation columns, and column temperatures were investigated. Within 15 min, these 28 metabolites were effectively separated using a HILIC column operated under gradient conditions with mobile phases consisting of water (containing 0.5% acetic acid and 10 mM ammonium acetate) and methanol (also containing 0.5% acetic acid). Figure 1 shows typical chromatographic profiles of these metabolites, which were generated using 0.01 mL of crude serum pool extract.

### Method validation

#### Linearity

After conducting 10 separate analytical runs, the regression equation between peak area ratios (y) of these 28 analyses to their internal standards and corresponding concentrations of them (x) has been identified. The results of the linearity study, including four linear parameters, are given in Table 3. The average linear correlation coefficients were 0.9989 and 0.9984 for Ile and C18 ceramide, respectively. The average linear correlation coefficients for the rest of the analytes were more than 0.999, suggesting good linearity.

**Table 3:** Calibration parameters of serum amino acids, ceramides, LPCs, C0 and AcyCNs by the HILIC-ESI-MS/MS method

Metabolites	Species	Slope (a)	Intercept (b)	Standard error of the estimate (y)	Correlation coefficient (*r^2^*)
Amino acids	Val	0.01	0.01	0.04	0.9994
	Ile	0.001	0.01	0.12	0.9989
	Leu	0.09	0.37	0.06	0.9995
	Tyr	0.01	−0.004	0.03	0.9993
	Phe	0.01	0.02	0.05	0.9993
	Trp	0.01	0.01	0.13	0.9992
	Glu	0.01	0.01	0.02	0.9995
	Gln	0.001	0.003	0.04	0.9993
Ceramides	C16	0.26	0.06	0.05	0.9999
	C18	0.31	0.82	0.06	0.9984
	C24	0.58	0.08	0.05	0.9996
LPCs	16:0	0.03	0.04	0.05	0.9997
	18:0	0.004	−0.0003	0.03	0.9998
	18:1	0.02	0.01	0.03	0.9998
C0 and AcyCNs	C0	0.01	−0.003	0.03	0.9994
	C2	0.06	−0.01	0.04	0.9996
	C3	0.20	−0.003	0.03	0.9997
	C4	2.56	−0.002	0.03	0.9997
	C5	0.09	−0.04	0.05	0.9993
	C6	11.98	−0.001	0.04	0.9997
	C8	0.59	0.004	0.05	0.9994
	C10	0.26	−0.003	0.03	0.9999
	C10:1	0.23	−0.004	0.04	0.9997
	C12	0.62	−0.0004	0.06	0.9998
	C14	14.01	−0.04	0.06	0.9996
	C16	2.73	0.02	0.05	0.9994
	C18	58.09	−0.01	0.04	0.9994
	C18:1	4.51	−0.32	0.18	0.9991

#### Recovery

For the recovery study, we added different mix standards to the serum. The average analytical recoveries of 28 analytes were 91.3–106.3%, with a mean of 99.5%, which is summarized in Table 4. However, although the analytical recoveries were nearly 100%, the internal standards’ absolute recoveries ranged from 71.7 to 118.2%, which suggested that matrix interferences were present.

**Table 4:** Analytical recoveries of the HILIC-ESI-MS/MS method

Metabolites	Species	Initial concentration (μmol/L)	Spiked standards (μmol/L)	Detected concentration (μmol/L)	Recovery (%)			Initial concentration (μmol/L)	Spiked standards (μmol/L)	Detected concentration (μmol/L)	Recovery (%)
Amino acids	Val	184.11	21.51	205.33	98.64	C0 and AcyCNs	C0	42.09	9.31	51.43	100.40
			107.55	292.57	100.85				46.53	88.63	100.02
	Ile	51.21	18.95	70.54	101.99		C2	6.68	1.02	7.71	101.06
			94.74	146.11	100.17				5.10	11.80	100.38
	Leu	80.43	18.82	99.90	103.47		C3	2.95 × 10^-1^	1.05 × 10^-1^	3.98 × 10^-1^	97.63
			94.1	175.31	100.83				5.25 × 10^-1^	8.10 × 10^-1^	98.16
	Tyr	51.25	13.97	65.20	99.85		C4	2.81 × 10^-1^	4.32 × 10^-2^	3.25 × 10^-1^	102.72
			69.85	121.16	100.08				2.16 × 10^-1^	4.99 × 10^-1^	101.06
	Phe	75.76	14.99	91.13	102.51		C5	8.18 × 10^-1^	9.10 × 10^-3^	8.27 × 10^-1^	96.94
			74.94	150.95	100.33				4.55 × 10^-2^	8.62 × 10^-1^	97.08
	Trp	51.67	12.27	63.34	95.05		C6	1.03 × 10^-1^	9.69 × 10^-3^	1.13 × 10^-1^	100.11
			61.37	113.32	100.46				4.85 × 10^-2^	1.51 × 10^-1^	99.81
	Glu	106.54	17.11	122.68	94.31		C8	1.21 × 10^-1^	4.04 × 10^-2^	1.61 × 10^-1^	98.39
			85.55	191.16	98.91				2.02 × 10^-1^	3.20 × 10^-1^	98.74
	Gln	531.88	68.64	598.88	97.61		C10	2.32 × 10^-1^	7.42 × 10^-2^	3.07 × 10^-1^	100.74
			343.19	876.03	100.28				3.71 × 10^-1^	6.03 × 10^-1^	100.03
Ceramides	C16	0.32	0.10	0.42	98.66		C10:1	2.29 × 10^-1^	1.37 × 10^-1^	3.66 × 10^-1^	100.06
			0.49	0.82	101.53				6.85 × 10^-1^	9.15 × 10^-1^	100.12
	C18	0.17	0.04	0.20	91.25		C12	8.53 × 10^-2^	3.20 × 10^-2^	1.16 × 10^-1^	96.18
			0.18	0.35	98.96				1.60 × 10^-1^	2.44 × 10^-1^	98.99
	C24	6.11	0.67	6.79	101.42		C14	3.68 × 10^-2^	8.43 × 10^-3^	4.58 × 10^-2^	106.27
			3.33	9.45	100.31				4.22 × 10^-2^	8.08 × 10^-2^	104.33
LPCs	16:0	119.38	10.46	129.39	95.76		C16	1.05 × 10^-1^	2.72 × 10^-2^	1.32 × 10^-1^	98.64
			52.29	170.75	98.24				1.36 × 10^-1^	2.41 × 10^-1^	99.74
	18:0	42.06	4.27	46.04	93.21		C18	1.93 × 10^-2^	7.30 × 10^-3^	2.67 × 10^-2^	101.47
			21.35	62.89	97.58				3.65 × 10^-2^	5.59 × 10^-2^	100.28
	18:1	25.36	2.27	27.62	99.36		C18:1	2.89 × 10^-1^	9.05 × 10^-2^	3.80 × 10^-1^	100.06
			11.37	37.05	102.75				4.53 × 10^-1^	7.42 × 10^-1^	100.01

#### Precision

Three frozen individual serums were tested in three replicates throughout three independent runs to investigate the precision of this assay. Total coefficient of variations (CVs) and intra-run CV were lower than 9.6 and 5.9% respectively for the measurement of all compounds. For amino acids and LPCs, which were high concentration metabolites, the total CVs and intra-run CV were less than 5.8 and 3.4% respectively. All these data are given in Table 5. These results demonstrated the reproducibility of the assay, and these serum metabolites were stable during the storage at −80°C.

**Table 5:** Imprecisions of the HILIC-ESI-MS/MS method

Metabolites	Species	Serum 1			Serum 2			Serum 3		
		Mean (μmol/L)	Intra-run CV (%)	Total CV (%)	Mean (μmol/L)	Intra-run CV (%)	Total CV (%)	Mean (μmol/L)	Intra-run CV (%)	Total CV (%)
Amino acids	Val	278.35	2.54	4.13	379.58	1.44	3.50	280.35	1.88	3.49
	Ile	77.4	1.65	3.21	121.12	3.12	4.59	106.2	3.33	4.02
	Leu	112.15	1.33	2.77	144.81	2.81	3.57	114.2	2.83	3.03
	Tyr	73.07	2.76	4.41	107.5	3.44	4.91	102.69	1.79	3.58
	Phe	89.28	1.32	2.93	97.32	2.02	3.21	118.6	2.54	3.08
	Trp	56.62	1.27	3.16	62.52	3.24	4.80	65.53	1.94	5.82
	Glu	141.58	1.78	2.54	117.46	1.64	3.38	128.56	1.20	2.36
	Gln	668.69	2.09	3.84	647.19	2.33	4.05	877.08	2.47	3.14
Ceramides	C16	0.29	2.57	4.80	0.38	2.49	3.55	0.42	2.83	3.10
	C18	0.14	4.97	9.26	0.19	3.63	8.39	0.15	4.16	7.92
	C24	4.43	2.52	4.20	5.05	2.99	3.51	6.32	2.78	4.74
LPCs	16:0	111.12	1.33	2.09	108.16	1.36	3.15	108.18	1.34	2.57
	18:0	42.52	1.76	2.60	37.07	1.93	3.37	45.91	2.25	3.68
	18:1	16.79	1.51	1.98	20.29	1.22	2.64	20.62	1.05	2.51
C0 and AcyCNs	C0	40.1	1.32	2.48	44.37	1.06	2.15	40.89	1.14	2.67
	C2	9.57	1.44	2.15	5.25	1.88	2.39	10.95	1.37	2.24
	C3	4.44 × 10^-1^	2.09	3.67	6.15 × 10^-1^	2.47	4.38	5.49 × 10^-1^	2.87	3.91
	C4	1.65 × 10^-1^	2.17	3.79	5.48 × 10^-1^	2.61	3.44	2.30 × 10^-1^	2.76	4.72
	C5	9.96 × 10^-2^	2.15	5.21	1.43 × 10^-1^	2.02	4.88	1.30 × 10^-1^	1.62	5.57
	C6	1.29 × 10^-1^	3.86	7.65	4.10 × 10^-2^	5.89	9.64	1.82 × 10^-1^	4.64	8.50
	C8	4.26 × 10^-1^	2.52	3.24	7.91 × 10^-2^	3.15	3.71	3.97 × 10^-1^	2.35	4.96
	C10	4.96 × 10^-1^	1.92	2.77	8.89 × 10^-2^	2.00	4.42	5.62 × 10^-1^	2.08	3.68
	C10:1	5.69 × 10^-1^	2.15	2.61	1.57 × 10^-1^	2.34	3.49	4.88 × 10^-1^	2.22	3.17
	C12	7.74 × 10^-2^	1.19	2.20	5.65 × 10^-2^	2.08	3.15	1.92 × 10^-1^	1.44	2.11
	C14	2.75 × 10^-2^	1.23	3.48	1.31 × 10^-2^	2.76	3.43	5.60 × 10^-2^	2.74	3.27
	C16	1.23 × 10^-1^	2.27	3.20	8.61 × 10^-2^	2.74	4.24	1.26 × 10^-1^	1.27	3.65
	C18	5.72 × 10^-2^	2.99	5.30	3.20 × 10^-2^	3.26	9.42	2.63 × 10^-2^	2.57	7.74
	C18:1	1.81 × 10^-1^	2.57	5.53	1.14 × 10^-1^	3.32	6.83	1.91 × 10^-1^	2.95	6.35

#### Limits of detection and quantification (sensitivity)

The LOD and LOQ values were obtained by serially diluting the lowest standard solution at known concentration until S/N = 3 and 10 were reached in 10 replicates. The determined LOD and LOQ levels of the target metabolites are given in Table 6. Based on our results, the serum concentrations of any of the target chemicals were significantly lower than reported (24, 27). The developed approach had sufficient sensitivity to quantify these substances in serum samples.

**Table 6:** LOD and LOQ of amino acids, ceramides, LPCs, C0 and AcyCNs

Metabolites	Species	LOD (μmol/L)	LOQ (μmol/L)
Amino acids	Val	2.48 × 10^-1^	8.27 × 10^-1^
	Ile	5.57	9.86
	Leu	1.64 × 10^-1^	5.17 × 10^-1^
	Tyr	1.76 × 10^-1^	5.47 × 10^-1^
	Phe	7.78 × 10^-2^	2.09 × 10^-1^
	Trp	1.38 × 10^-1^	4.51 × 10^-1^
	Glu	5.23 × 10^-1^	1.01
	Gln	1.03	3.43
Ceramides	C16	5.20 × 10^-3^	1.73 × 10^-2^
	C18	2.45 × 10^-3^	8.18 × 10^-3^
	C24	2.30 × 10^-2^	7.66 × 10^-2^
LPCs	16:0	3.78 × 10^-2^	1.01 × 10^-1^
	18:0	5.96 × 10^-2^	1.29 × 10^-1^
	18:1	4.01 × 10^-2^	8.76 × 10^-2^
C0 and AcyCNs	C0	1.96 × 10^-1^	6.12 × 10^-1^
	C2	1.60 × 10^-2^	5.23 × 10^-2^
	C3	6.29 × 10^-3^	1.47 × 10^-2^
	C4	8.76 × 10^-3^	2.72 × 10^-2^
	C5	3.28 × 10^-3^	6.59 × 10^-3^
	C6	2.91 × 10^-3^	4.69 × 10^-3^
	C8	4.12 × 10^-3^	9.48 × 10^-3^
	C10	5.19 × 10^-3^	1.63 × 10^-2^
	C10:1	8.98 × 10^-3^	2.19 × 10^-2^
	C12	1.20 × 10^-3^	3.07 × 10^-3^
	C14	6.13 × 10^-4^	1.43 × 10^-3^
	C16	5.83 × 10^-3^	1.04 × 10^-2^
	C18	1.62 × 10^-3^	4.08 × 10^-3^
	C18:1	1.97 × 10^-3^	3.56 × 10^-3^

## Discussion

Over the past decade, a series of published data have found that dietary intake is linked to an increased chance of developing MM and even its precursor disease (28, 29). At the same time, the levels of serum metabolites obtained from food and endogenous substances controlled by dietary consumption, such as C0, species of AcyCNs, amino acids, ceramides, and LPCs, consistently have been found altered in MM patients in some metabolomics research (30–32). These observations suggested serum diet-related metabolites have been identified as potential biomarkers for the progression of MM and may assist in evaluating MM risk. However, most of these investigations employed untargeted or targeted metabolomics strategies, thereby having some intrinsic limitations, namely low reproducibility and compromising the accuracy of the results (33, 34). Therefore, it would be necessary to develop a precise and reliable approach to validate the usefulness of these potential diary biomarkers in MM and make the different studies more comparable. This work established and validated a precise, accurate, specific, and efficient assay for parallel measurements of 28 metabolites in serum samples within 15 min.

In contrast to conventional analytical techniques, LC-MS/MS was chosen to serve as a platform for measuring these targeted metabolites in view of its remarkable sensitivity and selectivity, and it has the minimum requirements for sample preparation. In this study, compared to hydrophilic amino acids and short chain AcyCNs, long chain AcyCNs, LPCs and ceramides are mainly weak-polar metabolites, optimizing the chromatographic condition for separation and simultaneous analysis of all these compounds can be challenging. In our earlier research, we developed a precise assay for quantitation of C0, BCAAs, AAAs, Glu, Gln, LPCs, and three types of AcyCNs by HILIC column (35), but under such proportion of mobile phase with isocratic elution mode, obvious chromatographic peaks trailing of the ceramides were observed. Most of the existing LC-MS/MS methodologies of ceramides determination selected reversed phase columns because of their capacity to retain and separate lipophilic substances (23, 36). However, amino acids cannot have adequate retention on these columns because of their hydrophilic nature. In this work, after analyzing the effectiveness of several different columns in separating the metabolites, we selected the HILIC column again, and the gradient elution program was studied to improve the chromatographic resolution of these diverse polarities analytes. Based on our results, using gradient elution protocols, the target compounds were maintained, separated, and eluted with satisfactory repeatability from run to run.

Another critical consideration that must be taken into account was that the serum concentration of these circulating molecules varies widely; for example, the concentration of Gln was about 10,000 times higher than the lowest concentration of C12 and C18 AcyCNs. Decreasing temperature and ion spray voltage can avoid the overloading of amino acids with high levels; however, some long chain AcyCNs with low concentration cannot be detected under such MS conditions. For greater manipulation of MS settings and to ensure maximum sensitivity, we ran AcyCNs in a separate period II from amino acids, and the total chromatographic separation was accordingly divided into two periods based on the different retention times. In the first section, between 0 and 4 min, intensities of ions for amino acids and three ceramides were monitored, while in the second acquisition period, between 4 and 10 min, intensities of ions for LPCs, C0, and AcyCNs were detected. The optimal ion source parameters and compound-dependent parameters were set for the analytes in two periods.

To enable a reliable LC-MS/MS quantification method, using stable isotope labeling is the most accurate way to quantify trace compounds in complex serum matrices. Deuterated isotope internal standards were utilized to measure C0, 14 AcyCNs, three ceramides, and eight amino acids. Since there were no commercially available isotope internal standards, C10:0-D3 and C18:0-D3 AcyCNs were employed as internal standards for C10:1 and C18:1, respectively. LPC 19:0 was used as the internal standard for three LPCs because it has minimal physic-chemical differences from them and doesn’t exist in human serum. Additionally, in our previous study, when we quantitated three different LPCs using stable isotope labeled LPCs and LPC 19:0 as the internal standard, we found the results did not significantly differ from one another (37).

The effective extraction of diverse metabolites from serum can improve the accuracy of the method. In this study, we evaluated methanol, acetonitrile, and isopropanol for their ability to extract metabolites from serum samples. According to the results, we observed the highest MS intensities and recoveries of the metabolites while using isopropanol as the extraction solvent compared to methanol or acetonitrile. Thus, the solvent for protein precipitation and serum preparation was chosen to be isopropanol. For Glu, Gln, and Trp, it was noted that there might be some matrix existing; nevertheless, the accuracy of the approach was not compromised because corresponding isotope internal standards were used for the quantification. For all the metabolites, the analytical recovery rates ranged from 91.3 to 106.3%, with an average of 99.5%. Additionally, the method’s sensitivity was adequate to identify the chemicals in samples because the serum physiological concentrations of them were significantly higher than LOQ (24, 37, 38). Total CVs of this method ranged from 1.98 to 9.64%, which showed good repeatability of this assay. These findings revealed that the LC-MS/MS approach was precise and dependable in its analysis of 28 metabolites in serum samples.

The present paper described developing an LC-MS/MS based method for simultaneously quantifying 28 diet-related metabolites in serum samples with 15 min run time. The current method employed a simple protein precipitation process for sample preparation, gradient elution for chromatographic separation, and two periods of MRM for MS detection. The proposed method has some advantages over the assays currently available in the literature, including efficiency, satisfactory sensitivity, precise and requires low volume sample, making it a direct tool for dynamically monitoring the physical condition along MM disease and aiding in directing appropriate disease-specific evaluation and management.
